# Cynaropicrin Averts the Oxidative Stress and Neuroinflammation in Ischemic/Reperfusion Injury Through the Modulation of NF-kB

**DOI:** 10.1007/s12010-022-04060-x

**Published:** 2022-07-15

**Authors:** Tao Jin, Bing Leng

**Affiliations:** 1 Department of Interventional and Vascular Surgery, Affiliated Tenth People’s Hospital of Tongji University, Shanghai, China; 2 Department of Neurosurgery, Affiliated Huashan Hospital of Fudan University, No. 12, Wulumuqi Middle Road, Shanghai, 200040 China

**Keywords:** Cerebral ischemia, Stroke, Inflammation, Natural products and cynaropicrin

## Abstract

Cerebral ischemia and successive reperfusion are the prevailing cause of cerebral stroke. Currently cerebral stroke is considered to be one of the prior causes for high mortality, disability, and morbidity. Cynaropicrin, a sesquiterpene lactone, exhibits various pharmacologic properties and also has an anti-inflammatory property associated with the suppression of the key pro-inflammatory NF-κB pathway. The protective effect of cynaropicrin against oxidative stress and neuroinflammation during CIR injury through the modulation of NF-κB pathway was studied in the current investigation. The experimental rats split into 5 groups as sham-operated control group (group 1), middle cerebral artery occlusion (MCAO)-induced rats (group 2), MCAO rats treated with cynaropicrin (diluted in saline) immediately 2 h after MCAO with 5, 10, and 25 mg/kg administration orally were designated as groups 3, 4, and 5, respectively. In MCAO-induced animals, the severity of ischemic was evident by the elevated level nitrate, MDA, MMPs, inflammatory mediators, Bax, caspase-3, and NF-κB. The level of Nrf-2, antioxidant enzymes, Bcl-2, and IL-10 was reduced in the MCAO-induced animals. Treatment with cynaropicrin in dosage-based manner increased the level of antioxidant enzymes, IL-10, Nrf-2, and Bcl-2 in the animals which indicates the antioxidative effect of cynaropicrin. The level of nitrate, MDA, MMPs, proinflammatory cytokines, inflammatory mediators, Bax, caspase-3, and NF-κB was reduced in the rats treated with cynaropicrin in a dosage-based manner. Experimental animals treated with cynaropicrin in a dosage-dependent way showed a defensive mechanism against oxidative stress and neuroinflammation by inhibiting the NF-κB pathway.

## Introduction

A considerable number of studies revealed that cerebral stroke is the most prominent neuro condition that leads to high disabilities and deaths in adults. The most prevalent type of brain stroke is ischemic. In ischemic stroke, the formation of a clot in the primary cerebral arteries results in obstruction of blood flow to the cerebral cells and causes cell death. This condition leads to paralysis [1]. Age, gender, clinical history, and other risk factors contribute to the risk of stroke [2]. Other notable risk factors that lead to stroke include smoking, obesity, high blood pressure, and high cholesterol level [3]. Considering current medical equipment, technological advancements, and surgical therapies, ischemic stroke remains a major global concern.

Ischemic stroke occurs when blood circulation to a part of the brain suddenly decreases or stops, leading to the loss of cognitive performance. The ischemic stroke starts with significant localized hypoperfusion [4]. Ischemic stroke involves a complicated cycle of cellular and molecular mechanisms that are interrelated. The mechanisms include loss of cell ion homeostasis, free radical-mediated toxicity, energy failure, acidosis, generation of arachidonic acid products, increased intracellular calcium levels, cytokine-mediated cytotoxicity, and infiltration of leukocytes complement activation, disruption of the blood-brain barrier (BBB), excitotoxicity, and activation of glial cells [5]. Oxidative stress and inflammation conditions are mainly responsible for the etiology of ischemic stroke. The major mechanism of cell death in the region of cerebral infarction is an energy shortage induced by the loss of neurons capacity to generate ATP [6]. The action of Na+/K+ ATPase diminishes as a result the extracellular level of K+ elevates and variation in the flow of Na+, Ca2+, and Cl−. Due to cytotoxic edema, the build of Na+ and Ca2+ occurs leading to dysfunction of organelles, loss of membrane stability, and apoptosis [7]. The Ca2+ flow increases which result in the activation of lipases, proteases, and endonuclease. In addition, the generation of ROS is elevated rapidly which leads to cell apoptosis [7]. Microglia and astrocytes release chemokines, matrix metalloproteinases, and cytokines in response to the elevated level of ROS [8]. The matrix metalloproteinase damages the base plate which leads to the increased blood-brain barrier permeability and thereby occurrence of inflammation. Reactive nitrogen species (RNS) have critical cellular impacts including inhibiting crucial mitochondrial enzymes, DNA damage, and promoting transition pore formation in mitochondria [9].

Nuclear factor-E2-related factor 2 (Nrf2) is considered to be the major regulator of natural antioxidant defense. The major role of Nrf2 is to protect brain cells against ischemic injury. Following the onset of ischemic injury, the impairment of Nrf2 amplifies the degree of the cerebral infarct and the severity of neurological impairments [10]. The potent regulator of inflammation and essential for cell death control is NF-κB transcriptional activation pathway. The activation of NF-κB occurs as an immediate reaction to the onset of stroke, and it plays a key role in BBB breakdown, inflammation, and cell apoptosis [11].

Artichoke (*Cynara scolymus*) is one of the oldest plants which have potent medicinal values. The extracts have been used in ancient medicines. They have potent an anti-inflammatory effect. The significant bioactive phytochemicals in artichoke extract are cynaropicrin (Cyn), a sesquiterpene lactone that is involved in the activation of Nrf2 [12]. Previous studies reveal that cynaropicrin is involved in the inhibition of the NF-κB transcriptional activation pathway [13]. Cynaropicrin exhibits several activities including anti-inflammatory, anti-tumor, antioxidative, antiparasitic, antiphotoaging agent, and anti-hyperlipidemic [14].

The objective of this study is to analyze the neuroprotective effect of cynaropicrin against induced cerebral ischemia-reperfusion (CIR) injury in experimental animals via modulation of the NF-κB pathway. The present investigation also explains the action of cynaropicrin on brain edema, the water content in the brain, infarct volume, neuro deficit score, antioxidant parameters, MMPs, nitrate, MDA, pro-inflammatory cytokines, inflammatory mediators, Nrf-2, NF-κB, and apoptosis markers.

## Materials and Methods

### Experimental Animal Model

Ten-week-old Wistar rats of weight 125 ±15 g were procured and utilized for the current investigation. They were caged and maintained in the controlled environmental condition of temperature at 25 ±2 °C and humidity at 55 ± 5% with alternative 12-h light and dark cycle. The animals were allowed to have water and food. Prior permission was obtained from the Institutional Animal Ethical Committee to carry out surgery and followed the protocols as mentioned by the institutional guidelines.

### Induction of Focal Cerebral Ischemia-Reperfusion

To initiate focal cerebral ischemia, the intraluminal occlusion of the middle cerebral artery (MAO) was carried out by adopting the procedures from previous publications [15]. Initially, the experimental animals were anesthetized by intraperitoneal administration of 75 mg/kg of xylazine and ketamine. To maintain sterile conditions during the surgery, the area was disinfected with 75% ethyl alcohol, and the temperature of the area was maintained at 37 °C to maintain the body temperature of the experimental animals until the completion of the surgery process. To carry out MCAO, the incision was made on the central region of the neck and then the submandibular glands were split. The right submandibular glands were obtained along with the sternocleidomastoid muscle. From the vagus nerve, the right common carotid artery was identified and isolated. 3-0 silk thread was used to prepare loose collar sutures around the CCA. The left CCA was joined and the CCA was temporally blocked using a 3-0 silk thread. To carry out 24 h of reperfusion, the blockage on the CCA was detached. Control animals underwent similar surgical techniques without MCAO blockage [16]. The experimental rats were split into 5 groups with 12 animals per group. Control group (sham-operated) as group 1; middle cerebral artery occlusion-induced animals as group 2; exactly 2 h after MCAO, groups 3, 4, and 5 rats were orally administrated with 5, 10, 25 mg/kg of cynaropicrin (diluted in saline), respectively. After the completion of the experiment, the brain tissues were collected and processed by immersing the collected tissues of 2 mm thickness in 2% tetrazolium chloride solution for 10 min at room temperature and analyzed for CIR [15, 17].

### Estimation of Water Content in the Brain

A part of brain tissue was dissected from the experimental animals to diagnose cerebral edema. The weight of the tissue was measured to calculate the wet weight. To calculate the dry weight, the brain tissue was placed in an oven at a temperature of 95 °C for a duration of 48 h. The water content (%) in the brain was evaluated by utilizing the formula:

1 $$\mathrm{Water}\ \mathrm{content}\ \left(\%\right)=\left[\left( WW- DW\right)/ WW\right]\times 100$$

### Blood-Brain Barrier Permeability

Evans blue extravasation test was utilized to calculate the blood-brain permeability [18]. Before reperfusion, the experimental animals were intravenously administrated with 1 ml/kg of Evans blue. After 24 h of MCAO, the brains of the experimental animals were perfused with saline. At the end of the experiment, the weight of the brain tissue obtained from the sham-operated group and other groups was measured. Then the brain tissues were homogenized using 50% TCA at 10,000g for a duration of 30 min. The supernatant was obtained and the Evans blue was measured at a wavelength of 610 nm. Evans blue concentration was measured in terms of μg/g of tissue, which was employed to estimate the blood-brain barrier permeability.

### Assessment of Neurological Deficits

The neurological function deficit test in CIR rats was done every hour after the onset of MCAO and subsequently every day for 5 days till the completion of the trials. The assessment of motor and behavioral deficits following chemical injection has been expressed as a four-point neuro-score: (0) no impairments; [1] difficulty in fully extending the contralateral forelimb; [2] inability to extend the contralateral forelimb; and [3] mild circling to the contralateral side [19].

### Determination of Antioxidant Markers

The oxidative parameters and antioxidant enzymes were measured in the ipsilateral ischemic region of the brain. The brain tissue was homogenized in Tris buffer (1% Triton X-100, 10% glycerol, 150 mM NaCl, 50 mM Tris-HCl, pH 7.4, protease inhibitor mixture) for a duration of 20 min at a temperature of 4 °C and then centrifuged at 4 °C for 20 min at 10,000g. The obtained supernatant was used to measure the oxidative parameters including 8-hydroxy-2′-deoxyguanosine (8-OHdG), nitrate, malondialdehyde (MDA), and nitrite. The antioxidant enzymes including catalase (CAT), superoxide dismutase (SOD), glutathione peroxidase (GSH-Px), and glutathione (GSH) were estimated using the commercially available assay kits by following the protocol mentioned by the manufacturer.

### Estimation of Pro-inflammatory Cytokines and Chemokines

Commercially available assay kits were utilized to estimate the effect of cynaropicrin on the inflection of cytokine levels in CIR. The levels of cyclooxygenase-2 (COX-2), prostaglandin E2 (PGE2), tumor necrosis factor-alpha (TNF-α), interleukin-10 (IL-10), IL-6, and IL-1β in the brain tissue of control and cynaropicrin-administrated animals were estimated using commercial assay kits [20, 21].

### Estimation of MMP-2 and MMP-9 Levels

To determine the antigelatinolytic effect of cynaropicrin, the activity of gelatinase (MMP-9 and MMP-2) was estimated using commercial gelatinase assay kits. The striatum and cortex obtained from the brain of the control group and other groups were homogenized using calcium chloride (pH 7.5) and 20 mM of Tris-HCl at 4 °C for a duration of 1 min. The sample was centrifuged for a duration of 1 h at 10,000g and the obtained supernatant was utilized for the assessment of MMP-9 and MMP-2 activity (colorimetric assay kit was used) [22, 23].

### Nrf2 DNA Binding Assay

Commercially available colorimetric assay kits were used to evaluate the role of Nrf2 in the DNA binding process. The collected brain tissues were homogenized and obtained supernatant fraction containing the nuclear extract. The Nrf2-coated plates were treated with the nuclear extracts containing transcription factors. The efficiency of Nrf2 binding with nuclear extracts was evaluated and represented as a percentage compared to control using the antigen-specific primary and secondary antibodies given with the kit.

### Real-Time Polymerase Chain Reaction (RT-PCR) Analysis

Quantitative RT-PCR was used to determine the mRNA expression of certain genes that modulate apoptosis and the NF-kB pathway in CIR. The RNeasy Mini Kit (Qiagen, USA) was used to extract total RNA from the ischemic penumbras of rat brains, which was then quantified using a Nanodrop spectrophotometer. A commercial assay kit was used for the transcription of RNA into cDNA. qRT-PCR was performed for certain genes using SYBY green PCR kits and further, the genes were multiplied by utilizing RT-PCR. In Table 1, the reverse (R) and forward (F) primers used for the amplification of certain genes are listed. The CT values were used to quantify gene expression, and the fold increase was computed using the comparative CT technique with GAPDH expression levels as the endogenous control.

**Table 1:** Primers used in RT-qPCR

Gene	Primer	Sequence (5′-3′)	Annealing	Accession number
iNOS	F	GTTGCTGGAAGGTGTGTTGG	59	AJ230461.1
	R	CAGGGTAAGGGCTTGGTGAC		
Bcl-2	F	TCGCGACTTTGCAGAGATGT	58	NM_016993.1
	R	CAATCCTCCCCCAGTTCACC		
Bax	F	CAACATGGAGCTGCAGAGGA	59	NM_017059.2
	R	TAGAAAAGGGCAACCACCCG		
Caspase-3	F	GAGCTTGGAACGCGAAGAAA	56	NM_012922.2
	R	TTGCGAGCTGACATTCCAGT		
NF-κB	F	CAGACACCTTTGCACTTGGC	59	NM_001276711.1
	R	CTTGAGTAGGACCCCGAGGA		
Nrf-2	F	ATTTGTAGATGACCATGAGTCGC	59	NM_031789.2
	R	TGTCCTGCTGTATGCTGCTT		

### Statistical Analysis

Statistical significance was determined by Tukey’s post hoc multiple comparison test and ANOVA (GraphPad Prism 5.0 Software). The data is represented as mean ± S.D. A *p* value of less than 0.05 was deemed highly significant.

## Results

### Effect of Cynaropicrin on the Cerebral I/R Injury in Rats

**Fig. 1:**
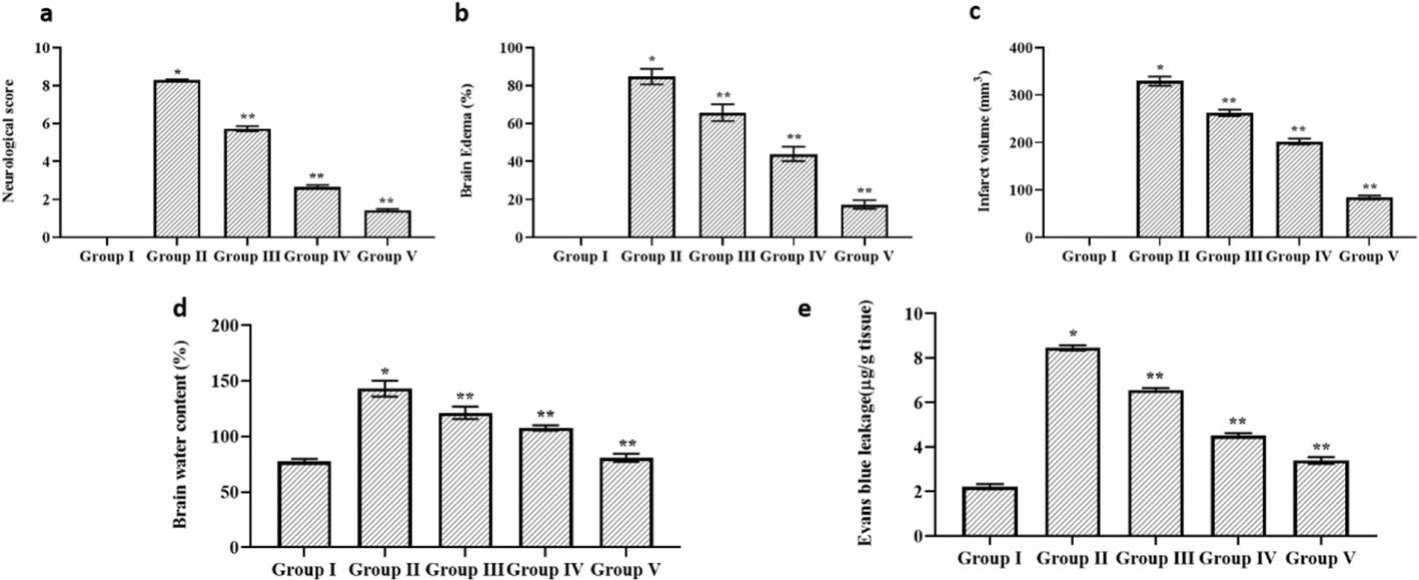
Effect of cynaropicrin on the cerebral I/R injury in rats. Cynaropicrin treatment effectively reduced the neurological scores (**a**), brain edema (**b**), brain infarct volume (**c**), brain water content (**d**), and Evans blue leakage (**e**) in the cerebral I/R injury-induced animals. Data are expressed as mean ± SD of triplicate values. *p < 0.05 when compared with the control group

Figure 1 depicts the impact of cynaropicrin on the cerebral I/R injury in rats. MCAO was performed to assess the protective impact of cynaropicrin against CIR. The neurological deficit score was calculated after 48 h of reperfusion. Figure 1a depicts the calculated neurological deficit score for the experimental animals. The score was amplified in the MCAO-induced group in comparison with cynaropicrin-treated groups. The brain edema, water content, and infarct volume were amplified in the MCAO-induced groups in comparison with drug-administrated groups. The blood-brain barrier permeability of the MCAO group was high. Upon cynaropicrin pre-treatment, the blood-brain barrier permeability decreased in the experimental animals. These results indicate that cynaropicrin acts effectively and secures the structure of the blood-brain barrier and neurological activities.

### Effect of Cynaropicrin on the Antioxidant Parameter in the Control and Cerebral I/R Injury in Rats

**Fig. 2:**
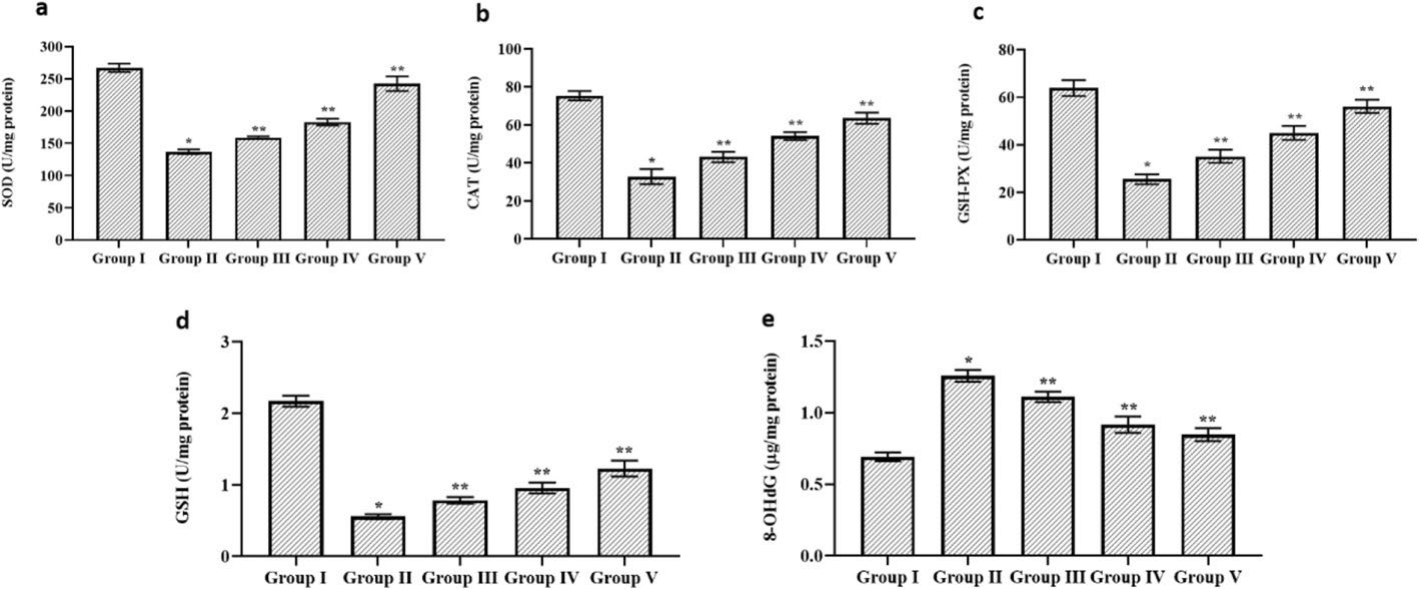
Effect of cynaropicrin on the antioxidant parameter in the control and cerebral I/R injury in rats. The cynaropicrin treatment appreciably elevated the superoxide dismutase (SOD) level (**a**), catalase (CAT) level (**b**), glutathione peroxidase (GSH-px) level (**c**), reduced glutathione (GSH) level (**d**), and decreased 8-hydroxy-2′-deoxyguanosine (8-OhdG) level (**e**) in the cerebral I/R injury-induced animals. Data are expressed as mean ± SD of triplicate values. *p < 0.05 when compared with the control group

Figure 2 depicts the effect of cynaropicrin on the antioxidant enzymes (SOD, CAT, GSH-PX, GSH, and 8-OhdG) in the control and cerebral I/R injury in rats. The level of SOD, CAT, GSH-PX, and GSH was decreased in the MCAO-induced group, and the level of these enzymes amplified with an increase in cynaropicrin dosage in groups 3, 4, and 5. In the control group, the level of SOD, CAT, GSH-PX, and GSH was normal. The activity level of 8-OhdG was elevated in the MCAO-induced rats in comparison with the control and cynaropicrin-pretreated group. This result reveals that cynaropicrin aids the antioxidants defense against oxidative stress.

### Effect of Cynaropicrin on the Nitrate, MDA, MMP-2, and MMP-9 Levels in the Control and Cerebral I/R Injury in Rats

**Fig. 3:**
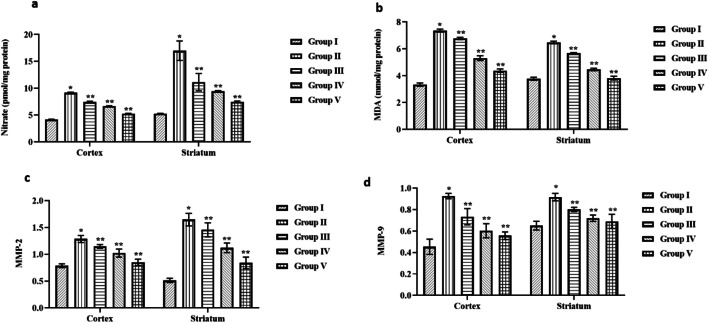
Effect of cynaropicrin the nitrate, MDA, MMP-2, and MMP-9 levels in the control and cerebral I/R injury in rats. Cynaropicrin treatment markedly downregulated the nitrate level (**a**), malondialdehyde level (**b**), matrix metalloproteinase-2 (MMP-2) level (**c**), and matrix metalloproteinase-9 (MMP-9) level (**d**) in both striatum and cortex of cerebral I/R injury-induced animals. Data are expressed as mean ± SD of triplicate values. *p < 0.05 when compared with the control group

Figure 3 shows the effect of cynaropicrin on the nitrate, MDA, MMP-2, and MMP-9 levels in the cortex and striatum of control and cerebral I/R injury in rats. The level of nitrate and MDA in the cortex and striatum region of cerebral tissue is elevated in the MCAO-induced group in comparison with the control and cynaropicrin-treated groups. The level of MMP-2 and MMP-9 in the cortex and striatum region of cerebral tissue of the MCAO-induced group is higher than the control and cynaropicrin-treated groups. This result reveals that cynaropicrin aids in the prevention of activation of MMP-9 and MMP-2 and also protects from lipid peroxidation.

### Effect of Cynaropicrin on the Pro-inflammatory Cytokines and Inflammatory Mediators in the Control and Cerebral I/R Injury in Rats

**Fig. 4:**
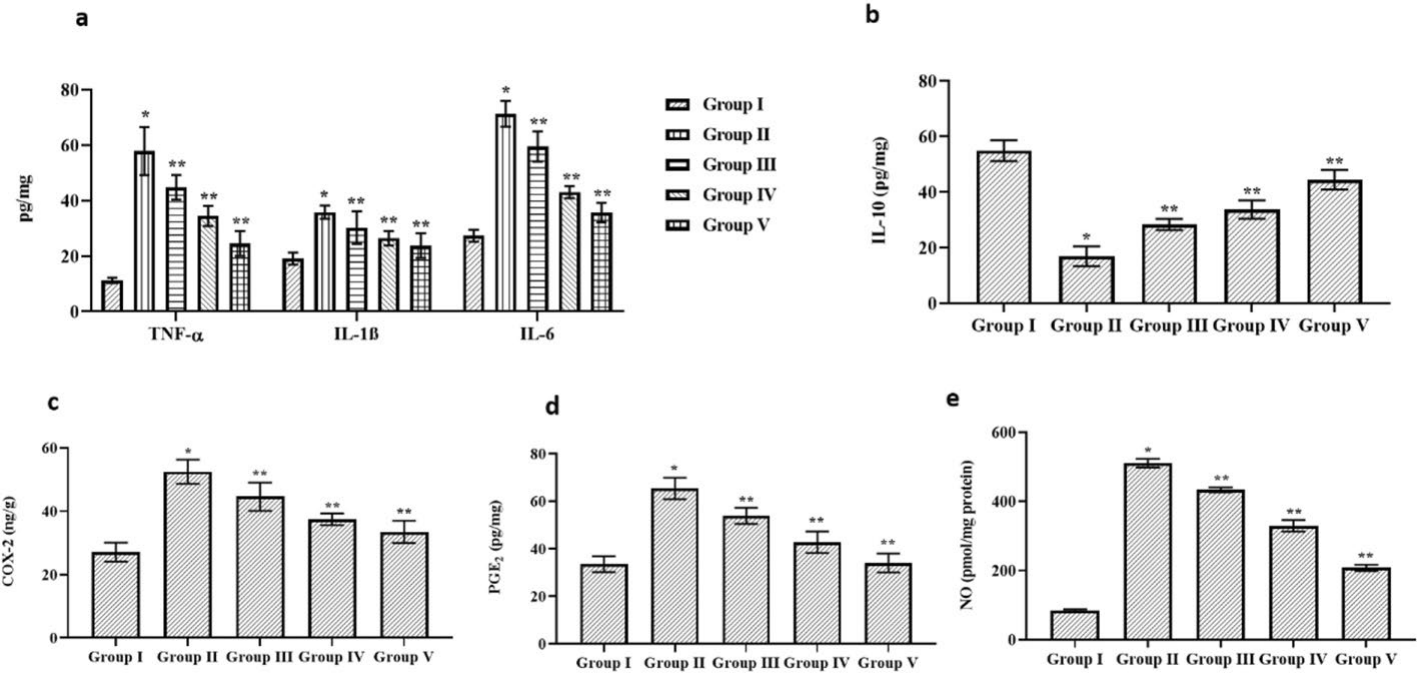
Effect of cynaropicrin on the pro-inflammatory cytokines and inflammatory mediators in the control and cerebral I/R injury in rats. Cynaropicrin treatment noticeably reduced the tumor necrosis factor-α (TNF-α), interleukin-1β (IL-1β), and interleukin-6 (IL-6) levels (**a**), increased interleukin-10 (IL-10) level (**b**), increased cyclooxygenase-2 (COX-2) level (**c**), prostaglandin-E2 (PGE2) level (**d**), and nitric oxide (NO) level (**e**) in the cerebral I/R injury-induced animals. Data are expressed as mean ± SD of triplicate values. *p < 0.05 when compared with the control group

Figure 4 demonstrates the effect of cynaropicrin on the pro-inflammatory cytokines (TNF-α, IL-10, IL-6, and IL-1β) and inflammatory mediators (COX-2, PGE2, and NO) in the control and cerebral I/R injury in rats. The activity level of TNF-α, IL-6, and IL-1β was upregulated in the MCAO-induced group in comparison to the control and dosage-based cynaropicrin-treated groups. The level of IL-10 was downregulated in MCAO-induced group in comparison to the control and cynaropicrin-administrated groups. The level of COX-2, PGE-2, and NO (inflammatory mediators) considerably increased in the MCAO-induced compared to the control and cynaropicrin-treated groups. Upon dosage-based cynaropicrin treatment, significantly reversed the increased level of inflammatory mediators.

### Effect of Cynaropicrin on the Apoptosis Marker in the Control and Cerebral I/R Injury in Rats

**Fig. 5:**
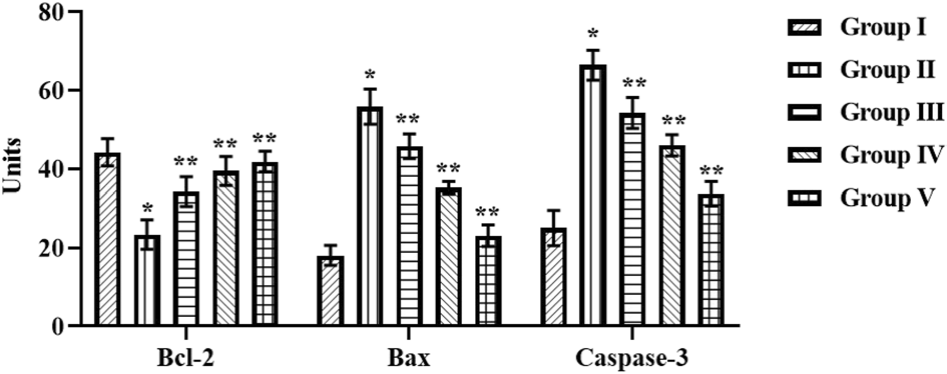
Effect of cynaropicrin on the apoptosis marker in the control and cerebral I/R injury in rats. As showed in the figure, the cynaropicrin treatment appreciably upregulated the mRNA expression of Bcl-2 and decreased the mRNA expressions of Bax and caspase-3 in the cerebral I/R injury-induced animals. Data are expressed as mean ± SD of triplicate values. *p < 0.05 when compared with the control group

Cynaropicrin acts on the apoptosis markers (Bcl-2, Bax, and caspase-3) in the control of cerebral I/R injury in rats (Fig. 5). The activity level of Bcl-2 was reduced in the MCAO-induced group. Upon dosage-based cynaropicrin treatment, the level of Bcl-2 was elevated similar to the control group. There was a significant increase in the level of caspase-3 and Bax gene level in the MCAO-induced group compared to the control and dosage-based cynaropicrin-treated group. This result indicates the positive impact of cynaropicrin against CIR injury.

### Effect of Cynaropicrin on the Nrf2 and NF-κB Activity in the Control and Cerebral I/R Injury-Induced Rats

**Fig. 6:**
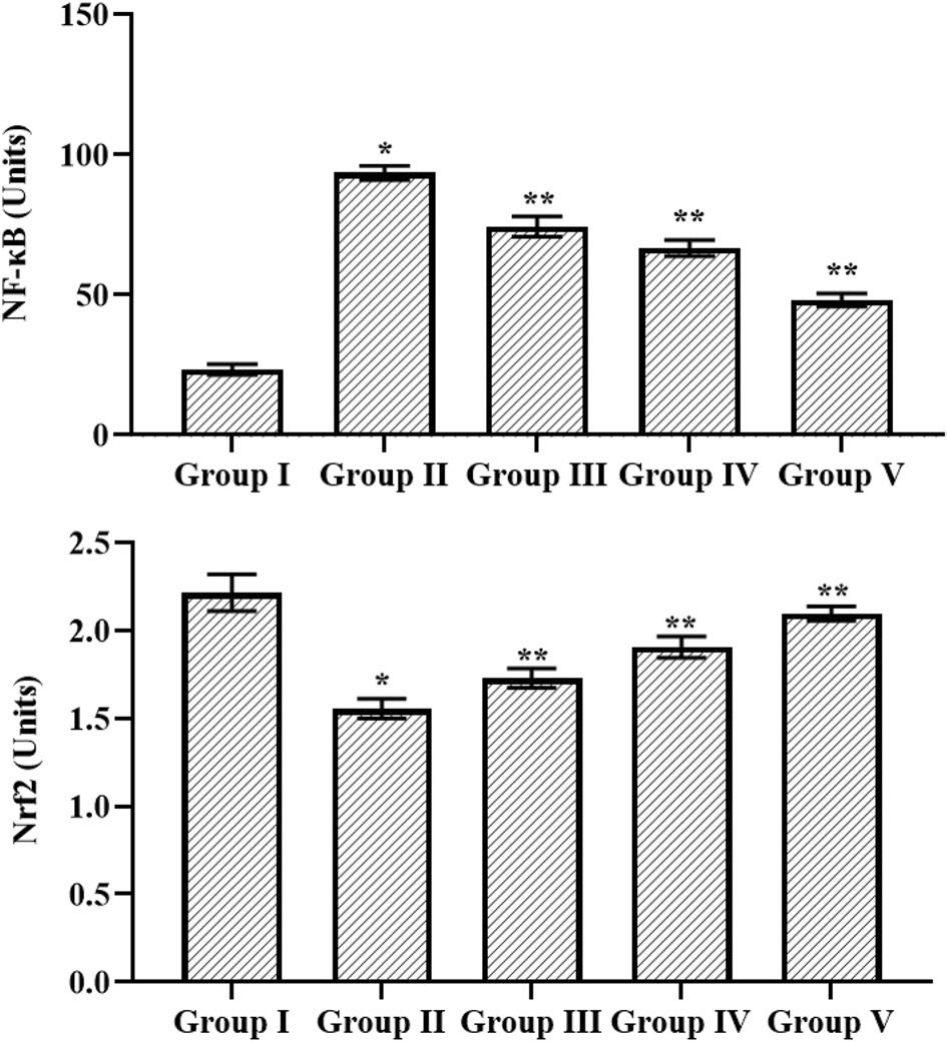
The effect of cynaropicrin on the Nrf2 and NF-κB activity in the control and cerebral I/R injury-induced rats. Treatment with cynaropicrin appreciably reduced the mRNA level of NF**-**κB and increased Nrf-2 level in the cerebral I/R injury-induced animals. Data are expressed as mean ± SD of triplicate values. *p < 0.05 when compared with the control group

Cynaropicrin has its effect on the Nrf2 and NF-κB activity in the control and cerebral I/R injury-induced rats (Fig. 6). The level of NF-κB was amplified in MCAO-induced group in comparison to other groups. Upon dosage-based treatment of cynaropicrin reversed the increased level of NF-κB in the experimental animals. There was a considerable decrease in the Nrf-2 level in the MCAO-induced groups than in other experimental animal groups. Animal groups treated with different dosages of cynaropicrin showed an increased level of cynaropicrin. This result reveals that cynaropicrin provides distinct defensive against CIR injury.

## Discussion

The present study utilized Wistar rats as experimental animal models to examine the protective effect of cynaropicrin against CIR injury. The MCAO induced in the experimental animals is identical to the ischemic stroke in humans. Stroke is caused due to oxidative stress and energy deprivation which leads to increased ROS production and onset of inflammation process [24]. Since the brain has a low supply of antioxidants, the neurons are more susceptible to oxidative stress than other body cells. The build-up of reducing intermediates caused by oxygen-glucose deprivation (OGD) leads to an electron leak, which creates free radicals and ROS [25]. The generated free radicals cause vascular damage, impairment of the blood-brain barrier followed by invariable cerebral damage. The mitochondrial membrane has depolarized as a result of ROS-induced oxidative stress, which might lead to apoptosis and accelerated aging of the cells [26]. Oxidative stress condition contributes to the DNA damage, depletion of ATP level, and lipid peroxidation which leads to injury of cells and tissues of the brain [27].

The neuroprotective effect of cynaropicrin was studied by inducing CIR in the animal model. Evaluation of water content, brain edema, infarct volume in the brain, BBB permeability, activity level of antioxidants, nitrate, MDA, MMP-2, MMP-9, proinflammatory cytokines, inflammatory mediators, apoptosis marker, Nrf-3, and NF-κB to assess the action of cynaropicrin.

There was a rise in the level of neuro deficit score, % edema, infarct volume, % water content in the brain, and permeability level of the blood-brain barrier in the MCAO-induced group as a result of cerebral I/R injury [28]. Reactive oxygen species plays a vital role in increasing the permeability of the blood-brain barrier which leads to tissue swelling followed by increased water content in the brain [29]. Treatment with cynaropicrin in dosage-based manner aids in the reduction of the edema, infarct volume, water content, and permeability of the blood-brain barrier which prevents the CIR injury.

SOD, CAT, GSH-PX, and GSH are considered being initial antioxidant enzymes. 8-OhdG is a critical biomarker of oxidative stress and DNA damage [30]. The antioxidant enzymes protect the cells from oxidative stress and ROS [31]. In MCAO-induced groups, the elevated level of antioxidant enzymes indicates the oxidative stress condition in the cerebral cells. The high level of 8-OhdG in the MCAO-induced group indicates the impact of DNA damage due to oxygen-free radicals. Treatment with cynaropicrin in a dosage-dependent way reduced the level of 8-OhdG in the experimental animals indicating the reduced DNA damage due to free radicals. There was an increase in the activity level of antioxidant enzymes in the animal groups which indicates the reduced oxidative stress condition. The antioxidative property of cynaropicrin leads to decreased oxidative stress and protected against CIR injury.

MMPs (matrix metalloproteinases) are vital biomarkers in CIR injury. In addition, MMPs play a crucial role in damaging the blood-brain barrier after cerebral stroke [32]. The primary enzymes for the breakdown of type IV collagen, which is the predominant component of the basement membrane, are gelatinase-A (MMP-2) and gelatinase-B (MMP-9). Previous studies suggest that suppression of MMPs resulted in decreased ischemic lesion effect indicating the activity of MMPs in CIR injury [33]. The final product of lipid peroxidation is malondialdehyde (MDA) considered to be a toxic end product and vital oxidative stress biomarker. Upregulation of MDA results in a significant elevation of ROS and a decrease in the level of antioxidants. Hike in MDA level can lead to cerebral cells damage [34]. The end products of NO are nitrate and nitrite. Nitrate is considered to be the biomarker of nitrosative damage. Previous studies indicate that the rise in nitrate level is due to the occurrence of CIR injury [35]. There was an elevation in the level of nitrate, MMPs, and MDA in the cortex and striatum of the MCAO-induced group. The rise in the level may be due to the increased free radicals which lead to oxidative stress and CIR injury. Administration of cynaropicrin in a dosage-dependent way to the experimental animals downregulated the activity level of these biomarkers indicating the action of cynaropicrin against the free radicals and nitrogen reactive species.

The vital proinflammatory cytokines are TNF-α, IL-10, IL-6, and IL-1β associated with cerebral stroke. The level of IL-6 is correlated to stroke intensity and treatment outcome. IL-6 is a crucial biomarker for evaluating the stroke severity [36]. TNF-α is a potent protein involved in inflammation and immune response activity. The level of TNF-α indicates the severity of neurological impairment [37]. Increased levels of TNF-α cause cell damage through the generation of free radicals [38]. IL-1 exists in two forms: IL-1α and IL-1β. After the onset of stroke, IL-1β activates other cytokines. The key biomarker for stroke is IL-1β [37]. IL-10 is an anti-inflammatory cytokine molecule that establishes a negative feedback system to restrict the expression of other proinflammatory cytokines [39]. In the present study, there was increased activity of TNF-α, IL-6, and IL-1β in the MCAO-induced animal group indicating the brain injury during the stroke. The level of TNF-α, IL-6, and IL-1β decreased in cynaropicrin-treated animals in a dose-dependent way. The protective effect of cynaropicrin reduced the activity of proinflammatory cytokines. The level of IL-10 is low in the MCAO-induced group indicating the inflammation of cells. The level of IL-10 was elevated in groups treated with cynaropicrin (dose-dependent) which shows the protective effect of cynaropicrin against inflammation.

The pro-inflammatory enzyme cyclooxygenase-2 (COX-2) is one of the inducible isoforms of cyclooxygenase (COX). Under normal brain conditions, the level of COX-2 is at a reduced level, but the level is altered by the activity of neurons [40]. Prostaglandin E2 (PGE2), a crucial product of COX-2, derived intermediate PGH2. In different neurological disorders, PGE2 is an important effector of the negative impacts mediated by inducible mPGES-1 and COX-2 [41]. Nitric oxide (NO) is a non-ionized gas that can readily pass through membranes. A set of three NO synthases (NOS) converts L-arginine to nitric oxide. In general, NO is inhibited by oxidation so the level of NO is low under normal brain conditions [42]. The level of COX-2, NO, and PGE2 in MCAO-induced group is elevated due to the occurrence of inflammation in the brain cells. On treatment with cynaropicrin in a dosage-dependent way, the level of COX-2, NO, and PGE2 was downregulated indicating the anti-inflammatory and oxidative property of cynaropicrin.

Caspase-3, Bcl-2, and Bax are crucial apoptotic proteins and are considered markers for the occurrence of brain injury. Caspase-3 controls neuronal cell death and is an essential protein in the apoptotic enzyme cascade [43]. Bcl-2 anti-apoptotic protein family is involved in activating or suppressing the intrinsic apoptotic pathway that is initiated by mitochondrial malfunction [20, 21]. The pro-apoptotic protein, Bax, regulates the apoptotic process and on activation causes cell apoptosis [22, 23]. The upregulated level of apoptotic proteins in the MCAO-induced group reveals neural apoptosis. Experimental animals treated with cynaropicrin in different dosages showed a decreased level of apoptotic proteins indicating the anti-apoptotic activity of cynaropicrin.

After cerebral ischemia, the NF-κB pathway extends the inflammatory response in the brain, activating inflammatory signals such as iNOS, COX-2, IL-1β, TNF-α, IL-6, and others [44]. Nrf-2 is the potent regulator of the cell defense system by regulating various cytoprotective genes [45]. Nrf-2 is involved in regulating ROS and the generation of antioxidant genes. Studies suggest that due to increased oxidative stress under-stroke condition results in downregulation of Nrf-2. MCAO-induced group showed a decreased level of Nrf-2. Cynaropicrin-treated groups with different dosages showed an increased level of Nrf-2. NF-κB is a transcription factor that plays a crucial role in increasing the production of antioxidative response components to counteract oxidative stress and inflammation. Under normal conditions, the level of NF-κB is very low. Upon CIR injury, the level of NF-κB increases. MCAO-induced group exhibits an increased level of NF-κB. Treatment with cynaropicrin in different doses to the experimental animals exhibits a reduced level of NF-κB. The increased level of Nrf-2 and decreased level of NF-κB in the drug-treated group reveal the neuroprotective effect of cynaropicrin.

## Conclusion

The present findings conclude that the cynaropicrin averts the oxidative stress and neuroinflammation in ischemic/reperfusion injury through the modulation of NF-kB. Cynaropicrin found to be an effective antioxidant and has an anti-inflammatory property which inhibited the reactive oxygen species thereby protected the neuronal cells from necrosis. Cynaropicrin aided in proper maintenance of proinflammatory enzymes, MDA, and antioxidant enzymes. Thus, cynaropicrin can serve as an effective therapeutic agent for CIR injury.

## Data Availability

Not applicable.
